# A new human-based metaheuristic algorithm for solving optimization problems on the base of simulation of driving training process

**DOI:** 10.1038/s41598-022-14225-7

**Published:** 2022-06-15

**Authors:** Mohammad Dehghani, Eva Trojovská, Pavel Trojovský

**Affiliations:** Department of Mathematics, Faculty of Science, University of Hradec Králové, Rokitanského 62, Hradec Králové, 500 03 Czech Republic

**Keywords:** Engineering, Mathematics and computing

## Abstract

In this paper, a new stochastic optimization algorithm is introduced, called Driving Training-Based Optimization (DTBO), which mimics the human activity of driving training. The fundamental inspiration behind the DTBO design is the learning process to drive in the driving school and the training of the driving instructor. DTBO is mathematically modeled in three phases: (1) training by the driving instructor, (2) patterning of students from instructor skills, and (3) practice. The performance of DTBO in optimization is evaluated on a set of 53 standard objective functions of unimodal, high-dimensional multimodal, fixed-dimensional multimodal, and IEEE CEC2017 test functions types. The optimization results show that DTBO has been able to provide appropriate solutions to optimization problems by maintaining a proper balance between exploration and exploitation. The performance quality of DTBO is compared with the results of 11 well-known algorithms. The simulation results show that DTBO performs better compared to 11 competitor algorithms and is more efficient in optimization applications.

## Introduction

Optimization is the process that determines the best solution to a problem with several feasible solutions. An optimization problem consists of three parts: decision variables, constraints, and the objective function^1^. In this case, the purpose of optimization is to quantify the decision variables with respect to the constraints of the problem so that the value of the objective function is optimized^2^. With the advancement of science and technology, the importance and role of optimization in various branches of science have become clearer. Therefore, practical tools are needed to address the various optimization challenges. Optimization techniques fall into two groups: deterministic and stochastic approaches. Deterministic approaches in both gradient-based and nongradient-based groups are effective in linear, convex, uncomplicated, low-dimensional, and differentiable problems. However, these approaches lose their effectiveness in dealing with optimization problems that have features such as nonlinear, nonconvex, complex, high-dimensional, notdifferentiable, discrete search space, and NP-hard problems. The difficulties and inefficiencies of deterministic approaches have led to the emergence of stochastic approaches that, using random operators, random search, and trial-and-error processes, are effective in optimization applications. Metaheuristic optimization algorithms, known as stochastic approaches, have become very popular and widely used due to advantages such as simple concepts, easy implementation, independent of the type of problem, no need for objective function-derived information, and efficiency in nonlinear, nonconvex environments, and nonlinear search space^3^. The optimization process in metaheuristic algorithms starts with generating a number of random candidate solutions in the range allowed for the search space. Then, in an iterative process, the candidate solutions are improved by the algorithm steps. After completion of the algorithm implementation iterations, the best candidate solution is introduced as the solution to the problem. The nature of random search in metaheuristic algorithms leads to the fact that there is no guarantee that this best candidate solution is the best solution (known as the global optimal) to a problem. Therefore, the best candidate solution is known as a quasi-optimal solution, which is an acceptable solution and close to the global optimal^4^. Achieving better quasi-optimal solutions has become a challenge in optimization studies to motivate researchers to introduce and design countless metaheuristic algorithms. In designing optimization algorithms, two indicators of exploration and exploitation play an important role in the performance of optimization algorithms in achieving appropriate quasi-optimal solutions. Exploring indicates the ability of the algorithm to perform a global search, and exploitation indicates the ability of the algorithm to perform a local search in the search space. The key to the success of a metaheuristic algorithm in the optimization process is maintaining a suitable balance between exploration and exploitation^5^. The main research question is whether, given that numerous optimization algorithms have been developed so far, is there still a need to design newer algorithms? The answer to this question, given the concept of No Free Lunch (NFL)^6^, is that there is no guarantee that an algorithm will work the same in all optimization problems. The NFL states that an algorithm may have a successful implementation on some optimization issues but fail to address others. Consequently, a particular algorithm cannot be considered the best optimizer for all optimization problems. Influenced by the concept of the NFL theorem, authors are encouraged to come up with more effective solutions to optimization problems by introducing new optimizers. The NFL theorem also motivated the authors of this paper to develop a new metaheuristic algorithm to address optimization applications. The novelty and contribution of this paper are in the design of a new metaheuristic algorithm called Driving Training-Based Optimization (DTBO), which is based on the simulation of human activity in driving education. The contributions of this paper are as follows:

- DTBO is introduced based on the driving training process in which a person is trained to learn driving skills.
- A set of 53 objective functions is used to analyze the performance of DTBO in optimization applications.
- To evaluate the quality of the performance of DTBO, the results obtained are compared with the results of 11 well-known optimization algorithms.
- The efficiency of DTBO is evaluated in solving two real-world applications.

The rest of the article is organized in such a way that in the “Lecture review”, the literature review is presented. In “Driving training based optimization”, the proposed DTBO approach is introduced and modeled. In “Computational complexity of DTBO”, simulation studies and results are presented. A discussion of the results and performance of the DTBO is provided in “Discussion. The application of DTBO in solving real-world problems is evaluated in the “DTBO for real-world applications”. The conclusions and several perspectives of the study are provided in “Conclusion and future works” section.

## Lecture review

Meta-heuristic algorithms have been developed inspired by various natural phenomena, wildlife, animals, birds, insects, plants, living organisms, laws of physics, biological sciences, genetics, rules of games, human activities, and other natural evolutionary processes. In a grouping based on the design’s primary inspiration, metaheuristic algorithms fall into five groups: swarm-based, evolutionary-based, physics-based, game-based, and human-based methods.

Swarm-based metaheuristic algorithms have been developed to model the swarming behaviors of animals, birds, and living things in nature. Among the famous algorithms that can be mentioned are Particle Swarm Optimization (PSO)^7^, Firefly Algorithm (FA)^8^, Artificial Bee Colony (ABC)^9^, and Ant Colony Optimization (ACO)^10^. The natural behavior of a group of birds or fish in search of food, while their movement is influenced by personal experience and swarming intelligence, has been the main idea in PSO design. Mathematical modeling of the natural feature of flashing lights in fireflies has been used in the FA design. The primary inspiration in ABC design is to simulate the intelligence of swarming bee colonies to find food sources. The ability of an ant colony to find the shortest path between the colony and food sources has been the main idea in the design of the ACO. Hunting and attacking prey strategy, as well as the process of finding food sources among living organisms, has been a source of inspiration in designing various metaheuristic algorithms such as the Tunicate Search Algorithm (TSA)^11^, Reptile Search Algorithm (RSA)^12^, Whale Optimization Algorithm (WOA)^13^, Orca Predation Algorithm (OPA)^14^, Marine Predator Algorithm (MPA)^15^, Pelican Optimization Algorithm (POA)^16^, Snow Leopard Optimization Algorithm (SLOA)^17^, Gray Wolf Optimization (GWO) algorithm^18^, Artificial Gorilla Troops Optimizer (GTO)^19^, African Vultures Optimization Algorithm (AVOA)^20^, Farmland Fertility^21^, Spotted Hyena Optimizer (SHO)^22^, and Tree Seed Algorithm (TSA)^23^.

Evolutionary-based metaheuristic algorithms have been introduced based on simulations of biological sciences, genetics, and using random operators. Among the most widely used and well-known evolutionary algorithms, you can name the Genetic Algorithm (GA)^24^ and Differential Evolutionary (DE)^25^. GA and DE have been developed on the basis of mathematical modeling of the reproductive process and the concept of natural selection, as well as the employment of random operators of selection, crossover, and mutation.

Physics-based metaheuristic algorithms are designed on the basis of mathematical modeling of various physical laws and phenomena. Among the well-known physics-based algorithms, one can mention the Simulated Annealing (SA)^26^ and the Gravitational Search Algorithm (GSA)^27^. SA is based on the physical phenomenon of melting and then cooling metals, known in metallurgy as annealing. The modeling of Gravitational Forces in a system consisting of objects with different masses and distances from each other has been the main inspiration in the design of GSAs. The physical phenomenon of the water cycle and its transformations in nature has been a source of inspiration for the design of the Water Cycle Algorithm (WCA)^28^. Cosmological concepts have been the main inspiration in the design of the Multi-Verse Optimizer (MVO)^29^. Some other physics-based methods are as follows: Flow Regime Algorithm (FRA)^30^, Nuclear Reaction Optimization (NRO)^31^, Spring Search Algorithm (SSA)^32^, and Equilibrium Optimizer (EO)^33^.

Game-based metaheuristic algorithms have been developed based on simulation of the rules that govern different games and the behavior of players, coaches, and other individuals who influence the games. The design of modeling competitions in the volleyball league has been the main idea in the design of the Volleyball Premier League (VPL) algorithm^34^ and the football league has been the main idea in the design of Football Game-Based Optimization (FGBO)^35^. The strategy and skill of the players to create puzzle pieces has been the main inspiration in designing the Puzzle Optimization Algorithm (POA)^36^. The effort of the players in tug-of-war was the main idea in designing the Tug-of-war Optimization (TWO) approach^37^.

Human-based metaheuristic algorithms are introduced on the basis of mathematical modeling of various human activities that have an evolution-based process. Teaching-Learning-Based Optimization (TLBO) is the most famous human-based algorithm designed based on simulation of the communication and interaction between a teacher and students in a classroom^38^. The economic activities of the rich and poor in society have been the main idea in designing Poor and Rich Optimization (PRO)^39^. Simulation of human behavior against online auction markets to achieve success has been used in the design of Human Mental Search (HMS)^40^. Interactions between doctors and patients, including disease prevention, check-up, and treatment, have been used in the design of DPO^41^.

Extensive studies have been conducted in the field of metaheuristic algorithms in various fields such as: development of binary versions^42–45^, improvement of existing methods^46–50^, and combination of metaheuristic algorithms^51,52^.

Based on the best knowledge gained from the literature review, so far, no optimization algorithm based on driving training modeling has been introduced and designed. The driving training process is an intelligent process that can be an incentive to design an optimizer. To address this research gap, in this paper, based on mathematical modeling of the driving training process and its various stages, a new metaheuristic algorithm is designed, which is introduced in the next section.

## Driving training based optimization

In this section, the various steps of the proposed Driving Training Based Optimization (DTBO) method are presented and then its mathematical modeling is introduced.

### Inspiration and main idea of DTBO

Driving training is an intelligent process in which a beginner is trained and acquires driving skills. A beginner as a learner driver can choose from several instructors when attending driving school. The instructor then teaches the learner driver the instructions and skills. The learner driver tries to learn driving skills from the instructor and drive following the instructor. In addition, personal practice can improve the driver’s skills of the learner. These interactions and activities have extraordinary potential for designing an optimizer. Mathematical modeling of this process is a fundamental inspiration in the design of DTBO.

### Mathematical model of DTBO

DTBO is a population-based metaheuristic whose members consist of driving learners and instructors. DTBO members are candidate solutions to the given problem modeled using a matrix called the population matrix in Eq. (1). The initial position of these members at the start of implementation is randomly initialized using Eq. (2).

1 $$\begin{aligned} X&= \begin{bmatrix} X_1 \\ \vdots \\ X_i \\ \vdots \\ X_N \end{bmatrix}_{N\times m} = \begin{bmatrix} x_{11} &{} \cdots &{} x_{1j} &{} \cdots &{} x_{1m} \\ \vdots &{} \ddots &{} \vdots &{} \ddots &{} \vdots \\ x_{i1} &{} \cdots &{} x_{ij} &{} \cdots &{} x_{im} \\ \vdots &{} \ddots &{} \vdots &{} \ddots &{} \vdots \\ x_{N1} &{} \cdots &{} x_{Nj} &{} \cdots &{} x_{Nm} \\ \end{bmatrix}_{N\times m}~, \end{aligned}$$

2 $$\begin{aligned} x_{i,j}&= lb_j + r\cdot (ub_j-lb_j), \quad i=1,2, \dots , N,\ j=1,2,\dots , m~, \end{aligned}$$

where *X* is the population of DTBO, $X_i$ is the *i*th candidate solution, $x_{i,j}$ is the value of the *j*th variable determined by the *i*th candidate solution, *N* is the size of the population of DTBO, *m* is the number of problem variables, *r* is a random number from the interval [0, 1], $lb_j$ and $ub_j$ are the lower and upper bounds of the *j*th problem variable, respectively.

Each candidate solution assigns values to the problem variables, which, by placing them in the objective function, are evaluated for the objective function. Therefore, a value is computed for the objective function corresponding to each candidate solution. The vector in Eq. (3) models the values of the objective function.

3 $$\begin{aligned} F = \begin{bmatrix} F_1 \\ \vdots \\ F_i \\ \vdots \\ F_N \end{bmatrix}_{N\times 1} = \begin{bmatrix} F(X_1) \\ \vdots \\ F(X_i) \\ \vdots \\ F(X_N) \end{bmatrix}_{N\times 1}~, \end{aligned}$$

where *F* represents the vector of the objective functions and $F_i$ denotes the value of the objective function delivered by the *i*th candidate solution.

The values obtained for the objective function are the main criteria to determine the goodness of the candidate solutions. Based on the comparison of the values of the objective function, the member that has the best value for the objective function is known as the best member of the population $(X_{best})$. The best member must also be updated, since the candidate solutions are improved and updated in each iteration.

The main difference between metaheuristic algorithms is the strategy employed in the process of updating candidate solutions. In DTBO, candidate solutions are updated in the following three different phases: (i) training the learner driver by the driving instructor, (ii) patterning the learner driver from instructor skills, and (iii) practice of the learner driver.

#### Phase 1: Training by the driving instructor (exploration)

The first phase of the DTBO update is based on the choice of the driving instructor by the learner driver and then the training of the driving by the selected instructor to the learner driver. Among the DTBO population, a select number of the best members are considered as driving instructors and the rest as learner drivers. Choosing the driving instructor and learning the skills of that instructor will lead to the movement of population members to different areas in the search space. This will increase the DTBO’s exploration power in the global search and discovery of the optimal area. Therefore, this phase of the DTBO update demonstrates the exploration ability of this algorithm. In each iteration, based on the comparison of the values of the objective function, the *N* members of the DTBO are selected as driving instructors, as shown in Eq. (4).

4 $$\begin{aligned} DI= \begin{bmatrix} DI_1 \\ \vdots \\ DI_i \\ \vdots \\ DI_{N_{DI}} \end{bmatrix}_{N_{DI}\times m} = \begin{bmatrix} DI_{11} &{} \cdots &{} DI_{1j} &{} \cdots &{} DI_{1m} \\ \vdots &{} \ddots &{} \vdots &{} \ddots &{} \vdots \\ DI_{i1} &{} \cdots &{} DI_{ij} &{} \cdots &{} DI_{im} \\ \vdots &{} \ddots &{} \vdots &{} \ddots &{} \vdots \\ DI_{N_{DI}1} &{} \cdots &{} DI_{N_{DI}j} &{} \cdots &{} DI_{N_{DI}m} \\ \end{bmatrix}_{N_{DI}\times m}~, \end{aligned}$$

where *DI* is the matrix of driving instructors, $DI_i$ is the *i*th driving instructor, $DI_{i,j}$ is the *j*th dimension, and $N_{DI} = \lfloor 0.1 \cdot N \cdot (1-t/T) \rfloor$ is the number of driving instructors, where *t* is the current iteration and *T* is the maximum number of iterations.

The mathematical modeling of this DTBO phase is such that, first, the new position for each member is calculated using Eq. (5). Then, according to Eq. (6), this new position replaces the previous one if it improves the value of the objective function.

5 $$\begin{aligned} x_{i,j}^{P1}&= {\left\{ \begin{array}{ll} x_{i,j} + r\cdot (DI_{k_i,j} - I\cdot x_{i,j} )~, &{} F_{DI_{k_i}} < F_i~; \\ x_{i,j} + r\cdot (x_{i,j} - DI_{k_i,j} )~, &{} \text {otherwise}~,\\ \end{array}\right. } \end{aligned}$$

6 $$\begin{aligned} X_{i}&= {\left\{ \begin{array}{ll} X_{i}^{P1} ~, &{} F_{i}^{P1} < F_i~; \\ X_{i}~, &{} \text {otherwise}~, \\ \end{array}\right. } \end{aligned}$$

where $X_i^{P1}$ is the new calculated status for the *i*th candidate solution based on the first phase of DTBO, $x_{i,j}^{P1}$ is its *j*th dimension, $F_i^{P1}$ is its objective function value, *I* is a number randomly selected from the set $\{1,2\}$, *r* is a random number in the interval [0, 1], $DI_{k_i}$, where $k_i$ is randomly selected from the set $\{1,2,\dots ,N_{DI}\}$, represents a randomly selected driving instructor to train the *i*th member, $DI_{k_i,j}$ is its *j*th dimension, and $F_{DI_{k_i}}$ is its objective function value.

#### Phase 2: Patterning of the instructor skills of the student driver (exploration)

The second phase of the DTBO update is based on the learner driver imitating the instructor, that is, the learner driver tries to model all the movements and skills of the instructor. This process moves DTBO members to different positions in the search space, thus increasing the DTBO’s exploration power. To mathematically simulate this concept, a new position is generated based on the linear combination of each member with the instructor according to Eq. (7). If this new position improves the value of the objective function, it replaces the previous position according to Eq. (8).

7 $$\begin{aligned} x_{i,j}^{P2}&= P\cdot x_{i,j} + (1-P)\cdot DI_{k_i,j}~, \end{aligned}$$

8 $$\begin{aligned} X_{i}&= {\left\{ \begin{array}{ll} X_{i}^{P2} ~, &{} F_{i}^{P2} < F_i; \\ X_{i}~, &{} \text {otherwise}~, \\ \end{array}\right. } \end{aligned}$$

where $X_i^{P2}$ is the new calculated status for the *i*th candidate solution based on the second phase of DTBO, $x_{i,j}^{P2}$ is its *j*th dimension, $F_i^{P2}$ is its objective function value, and *P* is the patterning index given by

9 $$\begin{aligned} P = 0.01 + 0.9 \left( 1-\frac{t}{T} \right) ~. \end{aligned}$$

#### Phase 3: Personal practice (exploitation)

The third phase of the DTBO update is based on the personal practice of each learner driver to improve and enhance driving skills. Each learner driver tries to get closer to his best skills in this phase. This phase is such that it allows each member to discover a better position based on a local search around its current position. This phase demonstrates the power of DTBO to exploit local search. This DTBO phase is mathematically modeled so that a random position is first generated near each population member according to Eq. (10). Then, according to Eq. (11), this position replaces the previous position if it improves the value of the objective function.

10 $$\begin{aligned} x_{i,j}^{P3}&= x_{i,j} + (1-2r)\cdot R\cdot \left( 1 - \frac{t}{T} \right) \cdot x_{i,j}~, \end{aligned}$$

11 $$\begin{aligned} X_{i}&= {\left\{ \begin{array}{ll} X_{i}^{P3} ~, &{} F_{i}^{P3} < F_i; \\ X_{i}~, &{} \text {otherwise}~, \\ \end{array}\right. } \end{aligned}$$

where $X_i^{P3}$ is the new calculated status for the *i*th candidate solution based on the third phase of DTBO, $x_{i,j}^{P3}$ is its *j*th dimension, $F_i^{P3}$ is its objective function value, *r* is a random real number of the interval [0, 1], *R* is the constant set to the value 0.05, *t* is the counter of iterations and *T* is the maximum number of iterations.

#### Repetition process, pseudo-Code of DTBO and DTBO flow chart

**Figure 1:**
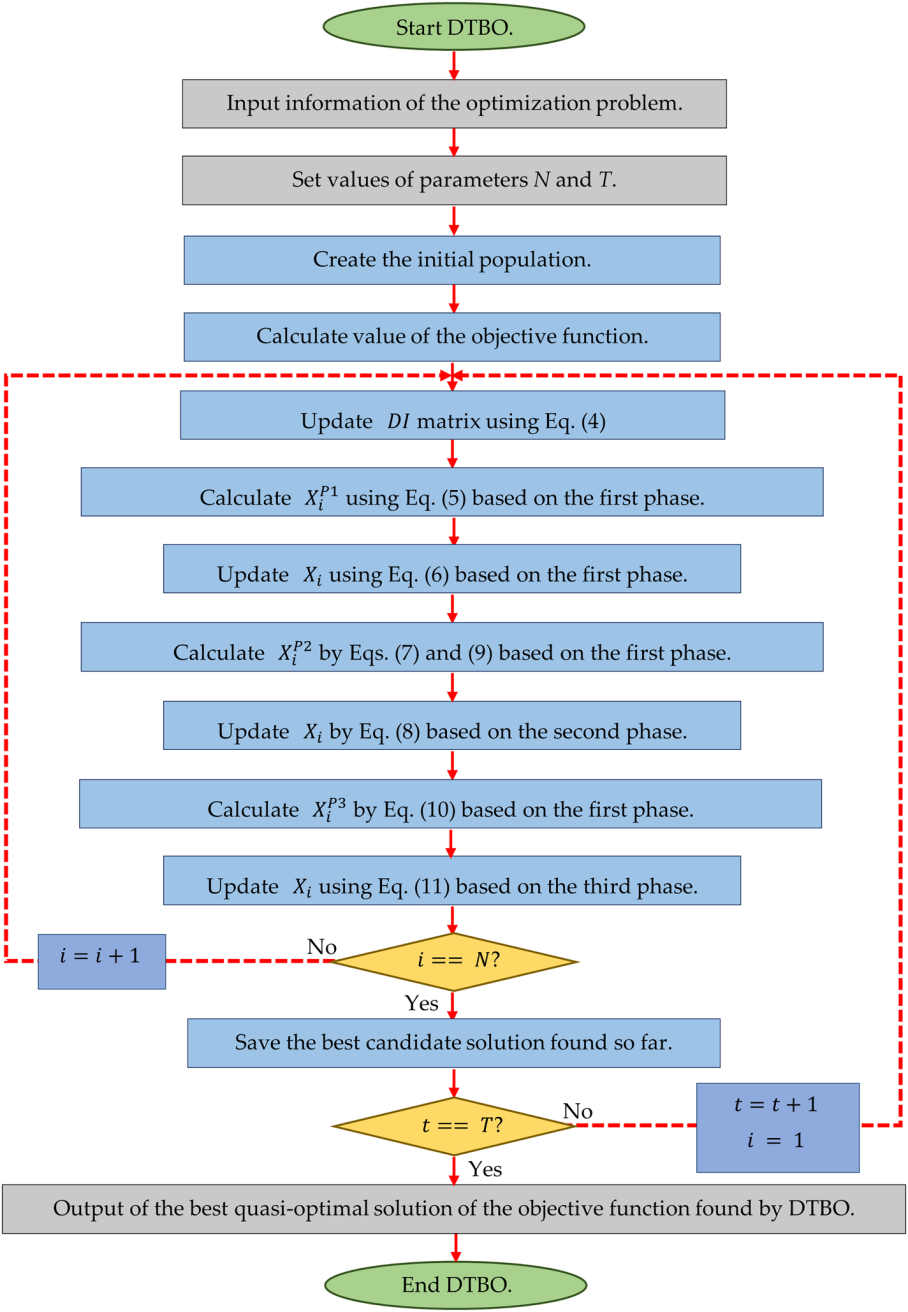
Flowchart of DTBO.

After updating the population members according to the first to third phases, a DTBO iteration is completed. The algorithm with the updated population entered the next DTBO iteration. The update process is repeated according to the steps of the first to third phases and according to Eqs. (4) to (11) to reach the maximum number of iterations. After the implementation of DTBO on the given problem is complete, the best candidate solution recorded during execution is introduced as the solution. The pseudocode of the proposed DTBO method is presented in Algorithm 1 and its flowchart is presented in Fig. 1.
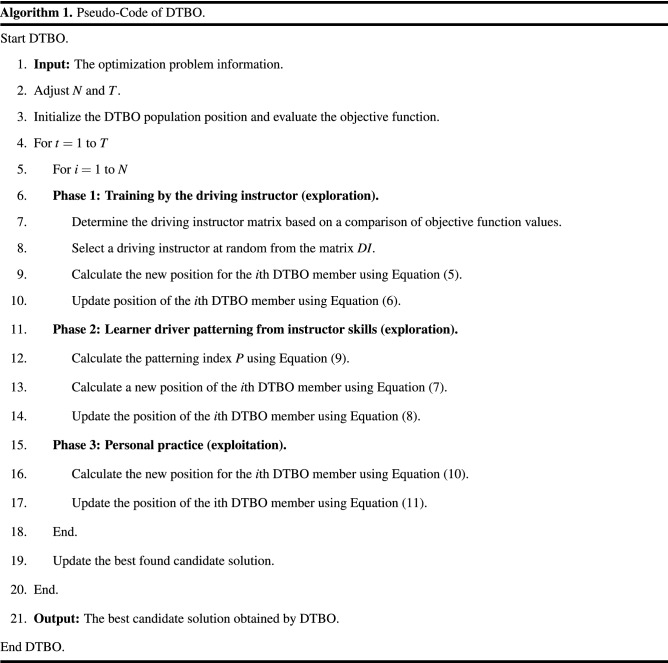

## Computational complexity of DTBO

In this subsection, we discuss the computational complexity of DTBO. The preparation and initialization of DTBO for the number of members equal to *N* and the problem with the number of decision variables equal to *m* have a computational complexity equal to ${O}(N\,m)$. In each iteration, the DTBO members are updated in three phases. Therefore, the computational complexity of the DTBO update processes is equal to ${O}(3N\,m\,T)$, where *T* is the maximum number of iterations of the algorithm. Consequently, the total computational complexity of DTBO is equal to ${O}(N\,m(1+3T))$.

### Simulation studies and results

This section is addressed to analyze the DTBO’s ability in optimization applications and provide optimal solutions to these types of problem. To this end, DTBO has been implemented on fifty-three standard objective functions of various types of unimodal, high-dimensional multimodal, fixed-dimensional multimodal^53^, and IEEE CEC2017 benchmark functions^54^. Furthermore, to evaluate the quality of the results obtained from DTBO, the performance of the proposed approach is compared with the performance of 11 well-known algorithms PSO, WOA, MVO, GA, GWO, GSA, MPA, TLBO, AVOA, RSA, and TSA. DTBO and competitor algorithms are used in twenty independent implementations, while each execution contains 1000 iterations to optimize the objective functions $F_1$ to $F_{23}$. The optimization results of the objective functions are reported using statistical indices mean, best, worst, standard deviation (std), median, and rank. The performance ranking criterion of optimization algorithms is based on the mean index. The values assigned to the control parameters of the competitor algorithms are listed in Table 1.

**Table 1:** Assigned values to the control parameters of competitor algorithms.

Algorithm	Parameter	Value
AVOA	Probability parameters	$P_1=0.6, P_2=0.4, P_3=0.6$
		$$(L_1,L_2)=(0.8,0.2)$$
	*w*	2.5
	$\beta$	1.5
	Random numbers	*h* is random number between $-2$ and 2
		*z* is random number between $-1$ and 1
		*u*, *v*, $rand_1,\dots ,rand_6$ are any random numbers between 0 and 1
RSA	Sensitive parameter	$\beta =0.01$
	Sensitive parameter	$\beta =0.1$
	Evolutionary sense (ES)	ES: randomly decreasing values between 2 and $-2$
MPA	Binary vector	$U = 0$ or $U = 1$
	Random vector	*R* is a vector of uniform random numbers in [0, 1]
	Constant number	$P = 0.5$
	Fish aggregating devices	$FADs=0.2$
TSA	$c_1, c_2, c_3$	Random numbers, which lie in the interval [0, 1]
	*Pmin*	1
	*Pmax*	4
WOA	$\ell$ is a random number in $[-1,1]$	
	*r* is a random vector in [0, 1]	
	Convergence parameter *a*	*a*: Linear reduction from 2 to 0
GWO	Convergence parameter *a*	*a*: Linear reduction from 2 to 0
MVO	Wormhole existence probability (WEP)	$Min(WEP) = 0.2$ and $Max(WEP)=1$
	Exploitation accuracy over the iterations (*p*)	$p=6$
TLBO	Random number	*rand* is a random number from the interval [0, 1]
	$T_F$: teaching factor	$T_F = \text {round}\, (1+rand)$
GSA	*Alpha*	20
	$G_0$	100
	*Rnorm*	2
	*Rnorm*	1
PSO	Velocity limit	10% of dimension range
	Topology	Fully connected
	Inertia weight	Linear reduction from 0.9 to 0.1
	Cognitive and social constant	$(C_1,C_2 )=(2,2)$
GA	Type	Real coded
	Mutation	Gaussian ($Probability = 0.05$)
	Crossover	Whole arithmetic ($Probability = 0.8$
	Selection	Roulette wheel (Proportionate)

### Evaluation of unimodal benchmark functions

The results of the implementation of DTBO and 11 competitor algorithms on the unimodal functions $F_1$ to $F_7$ are reported in Table 2. Comparison of statistical indicators shows that high-power DTBO has provided the global optimal in optimizing functions $F_1$, $F_2$, $F_3$, $F_4$, $F_5$, and $F_6$. Furthermore, DTBO performed better in optimizing the function $F_7$ and is the best optimizer for this function. Analysis of the simulation results shows that DTBO performs better in optimizing unimodal functions by providing far more competitive results than the other algorithms.

### Evaluation of high-dimensional multimodal benchmark functions

The optimization results of high-dimensional multimodal functions $F_8$ to $F_{13}$ using DTBO and 11 competitor algorithms are presented in Table 3. On the basis of the simulation results, it is evident that DTBO has made available the global optima of functions $F_9$ and $F_{11}$. DTBO is also the best optimizer for handling the functions $F_8$, $F_{10}$, $F_{12}$, and $F_{13}$. Comparing the performance of competitor algorithms against DTBO proves that DTBO, with its high ability, is much more efficient in optimizing multimodal functions.

### Evaluation of fixed-dimensional multimodal benchmark functions

**Figure 2:**
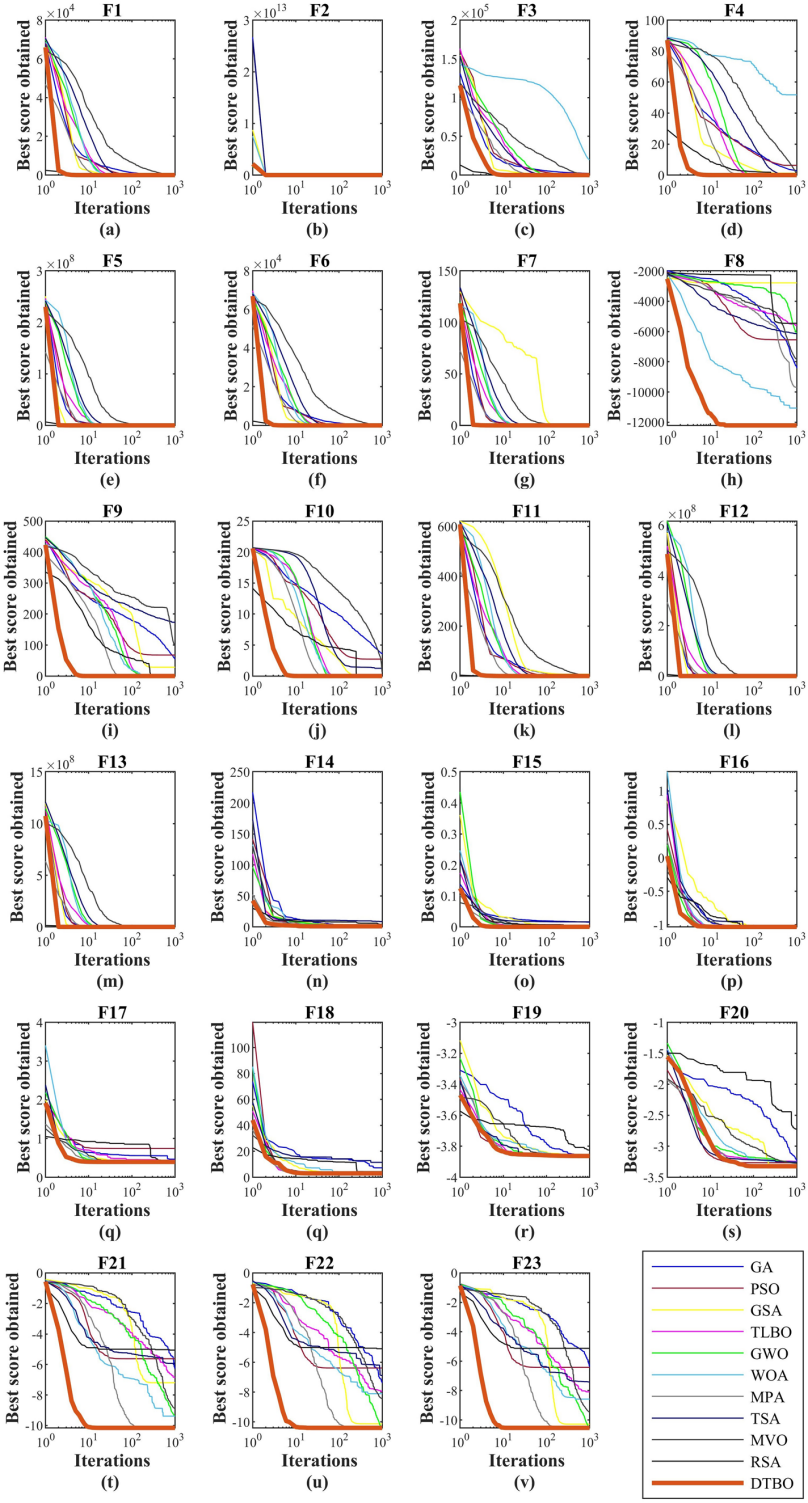
Convergence curves of DTBO and competitor algorithms in optimizing objective functions $F_1$ to $F_{23}$.

The optimization results obtained using DTBO and 11 competitor algorithms in optimizing fixed-dimensional multimodal functions from $F_{14}$ to $F_{23}$ are presented in Table 4. The optimization results show that DTBO is the best of all optimizers compared to handle all functions $F_{14}$ to $F_{23}$. Comparison of the performance of DTBO with competing algorithms shows that DTBO has effective efficiency and superior performance in handling fixed-dimensional multimodal functions. The behavior of the convergence curves of DTBO and competitor algorithms in achieving solutions for the objective functions $F_1$ to $F_{23}$ is presented in Fig. 2.

### Evaluation of IEEE CEC2017 benchmark functions

The results of the implementation of DTBO and competitor algorithms in the CEC 2017 benchmark functions, including 30 objective functions $C_1$ to $C_{30}$ are presented in Tables 5 and 6. What is clear from the optimization results is that DTBO has performed better in most CEC 2017 functions than competitor algorithms.

**Figure 3:**
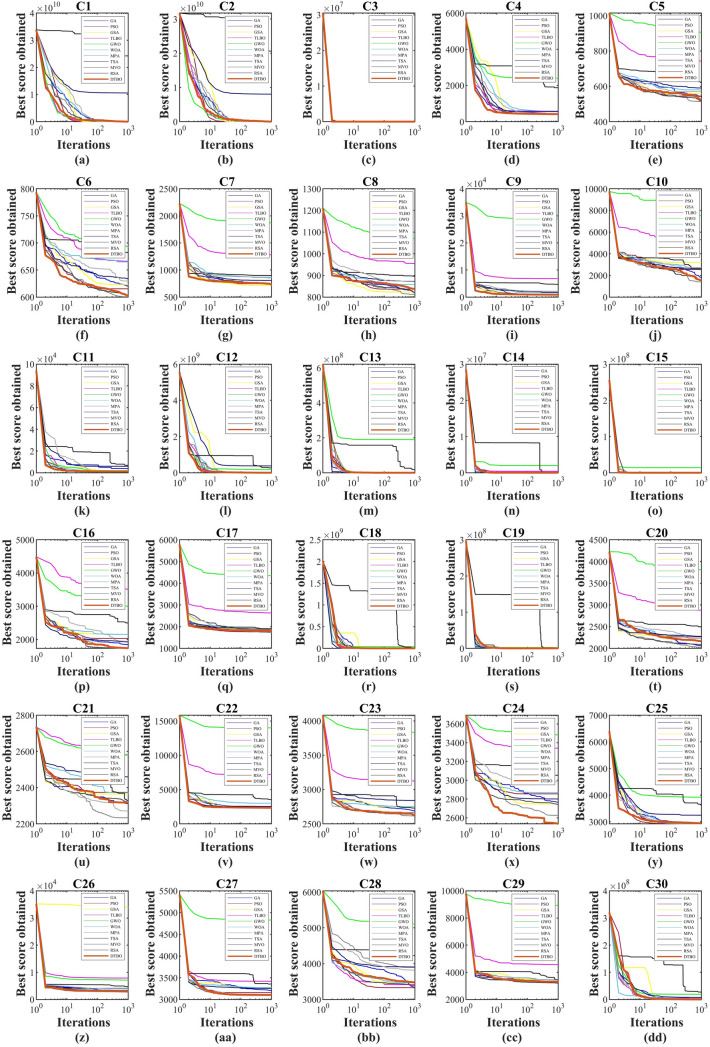
Convergence curves of DTBO and competitor algorithms in optimizing objective functions $C_1$ to $C_{30}$.

The convergence curves of DTBO and competitor algorithms while obtaining the solution for CEC2017 functions are shown in Fig. 3.

The analysis of the simulation results shows that the proposed approach in dealing with the CEC2017 benchmark functions, with acceptable results, has the first rank of the best optimizer, among the 11 algorithms compared.

### Statistical analysis

To provide statistical analysis of DTBO performance compared to competitor algorithms, the Wilcoxon sum rank test^55^ is used. The Wilcoxon sum rank test is a statistical test that, based on an indicator called the *p* value, shows whether the superiority of one method over another is statistically significant. The results of implementing the Wilcoxon sum rank test on DTBO in comparison with each of the competitor algorithms are presented in Table 7. Based on the results obtained, in each case where the *p* value is calculated less than 0.05, DTBO has a statistically significant superiority over the corresponding competitor algorithm.

**Table 2:** Evaluation results of unimodal functions.

		GA	PSO	GSA	TLBO	MVO	GWO	WOA	TSA	RSA	MPA	AVOA	DTBO
$F_1$	Mean	30.502	0.1010	1.3E$-$16	2.5E$-$74	0.1496	1.8E$-$59	1.4E$-$151	4.6E$-$47	1.9E$-$49	0	0	0
	Best	17.927	0.0005	5.4E$-$17	5.9E$-$77	0.1055	1.5E$-$61	9.3E$-$171	1.4E$-$50	3.8E$-$52	0	0	0
	Worst	56.928	1.3977	3.7E$-$16	2.6E$-$73	0.2013	7.7E$-$59	2.7E$-$150	3.3E$-$46	1.7E$-$48	0	0	0
	std	10.463	0.3108	7.1E$-$17	6.2E$-$74	0.0278	2.1E$-$59	6.0E$-$151	1.0E$-$46	3.9E$-$49	0	0	0
	Median	28.199	0.0097	1.1E$-$16	1.7E$-$75	0.1505	1.1E$-$59	2.2E$-$159	4.3E$-$48	4.2E$-$50	0	0	0
	Rank	10	8	7	3	9	4	2	6	5	1	1	1
$F_2$	Mean	2.7884	0.8955	5.5E$-$8	6.8E$-$39	0.2592	1.3E$-$34	2.5E$-$105	2.1E$-$28	7.0E$-$28	0	7.8E$-$281	0
	Best	1.7454	0.0453	3.5E$-$8	8.8E$-$40	0.1601	4.9E$-$36	7.9E$-$118	2.0E$-$30	1.8E$-$29	0	0	0
	Worst	3.8066	2.4933	1.2E$-$7	2.4E$-$38	0.3645	7.9E$-$34	2.7E$-$104	1.8E$-$27	4.7E$-$27	0	1.6E$-$279	0
	std	0.5448	0.7227	1.9E$-$8	5.6E$-$39	0.0630	2.0E$-$34	6.9E$-$105	5.3E$-$28	1.1E$-$27	0	0	0
	Med	2.7416	0.5842	5.1E$-$8	5.0E$-$39	0.2683	6.5E$-$35	3.4E$-$108	2.0E$-$29	3.5E$-$28	0	4.8E$-$298	0
	Rank	11	10	8	4	9	5	3	6	7	1	2	1
$F_3$	Mean	2169.0	388.13	475.50	3.8E$-$24	15.973	2.2E$-$14	19959.	1.2E$-$10	2.5E$-$12	0	0	0
	Best	1424.2	21.768	245.96	2.2E$-$29	5.9743	2.4E$-$19	2064.9	1.4E$-$21	6.2E$-$19	0	0	0
	Worst	3458.9	1025.4	1186.3	3.6E$-$23	48.940	4.1E$-$13	34688.	2.0E$-$9	1.4E$-$11	0	0	0
	std	639.69	288.43	220.28	1.1E$-$23	10.765	9.0E$-$14	8557.1	4.4E$-$10	4.4E$-$12	0	0	0
	Median	2100.7	293.04	400.33	4.0E$-$26	11.879	4.7E$-$16	20324.	1.1E$-$13	1.8E$-$13	0	0	0
	Rank	9	7	8	2	6	3	10	5	4	1	1	1
$F_4$	Mean	2.8294	6.2799	1.2359	1.8E$-$30	0.5471	1.2E$-$14	51.821	0.0044	3.0E$-$19	0	1E$-$269	0
	Best	2.2165	2.2903	9.9E$-$09	5.8E$-$32	0.2659	6.5E$-$16	0.9046	9.6E$-$05	3.02E$-$20	0	0	0
	Worst	3.9927	13.360	4.9277	8.1E$-$30	0.9630	5.7E$-$14	91.710	0.0358	9.6E$-$19	0	2E$-$268	0
	std	0.4669	2.5024	1.3871	2.4E$-$30	0.1922	1.5E$-$14	29.615	0.0079	2.3E$-$19	0	0	0
	Med	2.7835	5.8825	0.9069	6.5E$-$31	0.5310	6.3E$-$15	55.424	0.0015	2.6E$-$19	0	1.9E$-$283	0
	Rank	9	10	8	3	7	5	11	6	4	1	2	1
$F_5$	Mean	595.38	4611.9	44.050	26.788	96.222	26.582	27.310	28.477	23.324	4.3483	2.43E$-$05	0
	Best	228.81	26.281	25.885	25.589	27.632	25.567	26.722	25.671	22.809	8.8E$-$29	1.57E$-$06	0
	Worst	2257.1	901.28	167.2442	28.753	377.90	27.156	28.735	28.892	24.0493	28.990	7.37E$-$05	0
	std	424.99	20117.	44.323	0.9363	101.46	0.5263	0.5777	0.7881	0.3886	10.620	1.77E$-$05	0
	Median	475.57	86.098	26.346	26.328	30.018	26.232	27.087	28.823	23.295	9.7E$-$29	1.73E$-$05	0
	Rank	11	12	9	6	10	5	7	8	4	3	2	1
$F_6$	Mean	34.147	0.0634	1.1E$-$16	1.2614	0.1510	0.6608	0.0816	3.6820	1.8E$-$09	6.6156	3.92E$-$08	0
	Best	15.612	1.9E$-$6	5.52E$-$17	0.2331	0.0792	0.2467	0.0105	2.5528	8.1E$-$10	2.9073	2.34E$-$09	0
	Worst	62.767	0.5417	1.8E$-$16	2.1648	0.2501	1.2523	0.3267	4.7877	4.80E$-$09	7.4383	1.07E$-$07	0
	std	13.550	0.1486	3.7E$-$17	0.4972	0.0474	0.3066	0.1016	0.6934	9.4E$-$10	1.0998	2.61E$-$08	0
	Med	31.682	0.0021	9.5E$-$17	1.2174	0.1602	0.7273	0.0317	3.7960	1.6E$-$9	7.1097	3.33E$-$08	0
	Rank	12	5	2	9	7	8	6	10	3	11	4	1
$F_7$	Mean	0.0106	0.1841	0.0528	0.0015	0.0116	0.0008	0.0013	0.0043	0.0006	4.5E$-$5	0.000169	1.1E$-$5
	Best	0.0030	0.0690	0.0141	9.0E$-$05	0.0040	0.0002	2.0E$-$05	0.0015	0.0001	3.4E$-$6	6.55E$-$06	2.1E$-$6
	Worst	0.0219	0.4113	0.0956	0.0029	0.0226	0.0020	0.0054	0.0010	0.0009	0.0002	0.000739	3.4E$-$5
	std	0.0048	0.0790	0.0250	0.0009	0.0050	0.0005	0.0014	0.0023	0.0002	4.8E$-$5	0.000193	8.9E$-$6
	Median	0.0102	0.1777	0.0518	0.0015	0.0113	0.0008	0.0008	0.0037	0.0005	3.6E$-$5	9.3E$-$05	7.7E$-$6
	Rank	9	12	11	7	10	5	6	4	4	2	3	1
Sum rank		71	64	53	34	58	35	45	49	31	20	15	7
Mean rank		10.1429	9.14286	7.5714	4.8571	8.2857	5	6.4286	7	4.4286	2.8571	2.1429	1
Total rank		12	11	9	5	10	6	7	8	4	3	2	1

**Table 3:** Evaluation results of high-dimensional multimodal functions.

		GA	PSO	GSA	TLBO	MVO	GWO	WOA	TSA	RSA	MPA	AVOA	DTBO
$F_{8}$	Mean	− 8 421.5	− 6547.4	− 2781.3	− 5598.4	− 7833.0	− 6079.6	− 11065.1	− 6139.2	− 9687.5	− 5455.63	− 10317.6	− 12214.2
	Best	− 9 681.2	− 8244.2	− 3974.4	− 7028.1	− 9188.2	− 6863.4	− 12569.5	− 7319.0	− 10475.5	− 5707.92	− 10474.6	− 12569.5
	Worst	− 7 029.0	− 4989.0	− 2148.3	− 4550.0	− 6879.6	− 5048.0	− 7740.10	− 4369.9	− 9090.7	− 4906.74	− 8874.13	− 9016.3
	std	641.22	748.52	495.55	609.13	728.45	481.88	1735.10	729.88	370.23	258.77	434.05	1093.6
	Median	− 8399.1	− 6693.1	− 2693.0	− 5613.7	− 7710.8	− 6072.8	− 12040.8	− 6097.6	− 9719.5	− 5543.56	− 10474.6	− 12569.5
	Rank	5	7	12	10	6	9	2	8	4	11	3	1
$F_{9}$	Mean	54.6812	67.714	28.506	0	97.830	1.7E$-$14	0	173.12	0	0	0	0
	Best	23.232	39.798	13.929	0	52.787	0	0	89.745	0	0	0	0
	Worst	76.9009	114.56	48.753	0	149.28	1.1E$-$13	0	288.18	0	0	0	0
	std	13.808	18.841	9.1661	0	25.197	3.3E$-$14	0	51.007	0	0	0	0
	Med	52.6144	65.069	26.366	0	97.083	0	0	166.68	0	0	0	0
	Rank	4	5	3	1	6	2	1	7	1	1	1	1
$F_{10}$	Mean	3.5751	2.7272	8.2E$-$09	4.4E$-$15	0.5779	1.7E$-$14	4.1E$-$15	1.2425	4.3E$-$15	8.9E$-$16	8.9E$-$16	8.9E$-$16
	Best	2.8820	1.6934	4.7E$-$09	4.4E$-$15	0.1006	8.0E$-$15	8.9E$-$16	8.0E$-$15	8.9E$-$16	8.9E$-$16	8.9E$-$16	8.9E$-$16
	Worst	4.6420	5.0571	1.5E$-$08	4.4E$-$15	2.5152	2.2E$-$14	8.0E$-$15	3.3735	4.4E$-$15	8.9E$-$16	8.9E$-$16	8.9E$-$16
	std	0.3966	0.8578	2.3E$-$09	0	0.6772	3.6E$-$15	2.3E$-$15	1.5695	7.9E$-$16	0	0	0
	Median	3.6296	2.7339	7.7E$-$09	4.4E$-$15	0.1943	1.5E$-$14	4.4E$-$15	2.2E$-$14	4.4E$-$15	8.9E$-$16	8.9E$-$16	8.9E$-$16
	Rank	10	9	6	4	7	5	2	8	3	1	1	1
$F_{11}$	Mean	1.4735	0.1853	7.2080	0	0.3997	0.0013	0	0.0088	0	0	0	0
	Best	1.2881	0.0024	2.9956	0	0.2541	0	0	0	0	0	0	0
	Worst	1.7259	0.8758	12.638	0	0.5360	0.0188	0	0.0205	0	0	0	0
	std	0.1239	0.2285	2.7209	0	0.0819	0.0045	0	0.0063	0	0	0	0
	Med	1.4477	0.1224	7.3111	0	0.4165	0	0	0.0090	0	0	0	0
	Rank	6	4	7	1	5	2	1	3	1	1	1	1
$F_{12}$	Mean	0.2749	1.5011	0.2100	0.0713	0.9146	0.0399	0.0201	5.7928	2.0E$-$10	1.2763	3.9E$-$09	2.5E$-$14
	Best	0.0608	0.0001	4.70E$-$19	0.0241	0.0010	0.0126	0.0012	1.0369	5.2E$-$11	0.7294	1.0E$-$09	1.6E$-$32
	Worst	0.6508	5.2192	0.9318	0.1351	3.8480	0.0868	0.1369	14.136	3.8E$-$10	1.6297	1.0E$-$08	4.9E$-$13
	std	0.1386	1.2856	0.3074	0.0210	1.1967	0.0213	0.0400	3.8804	9.6E$-$11	0.2980	2.4E$-$09	1.0E$-$13
	Median	0.2644	1.2853	0.0802	0.0687	0.4203	0.0379	0.0058	4.3049	2.1E$-$10	1.1061	3.4E$-$09	1.6E$-$32
	Rank	8	11	7	6	9	5	4	12	2	10	3	1
$F_{13}$	Mean	2.7078	3.6076	0.0567	1.1020	0.0328	0.5138	0.2146	2.7169	0.0025	0.1636	1.0E$-$08	7.2E$-$13
	Best	1.2920	0.0096	4.7E$-$18	0.5885	0.0064	4.7E$-$05	0.0372	2.0125	0.0000	5.7E$-$32	4.2E$-$10	1.4E$-$32
	Worst	3.9402	12.586	0.9584	1.5412	0.0916	0.9501	0.7003	3.7139	0.0253	2.6729	3.6E$-$08	1.5E$-$11
	std	0.7545	3.0310	0.2136	0.2314	0.0248	0.2578	0.1835	0.5575	0.0063	0.6056	8.8E$-$09	3.2E$-$12
	Med	2.8672	3.3058	1.8E$-$17	1.1146	0.0236	0.5172	0.1658	2.5352	2.8E$-$09	5.1E$-$31	7.9E$-$09	1.4E$-$32
	Rank	10	12	5	9	4	8	7	11	3	6	2	1
Sum rank		43	48	40	31	37	31	17	49	14	30	11	6
Mean rank		7.1667	8	6.6667	5.1667	6.1667	5.1667	2.8333	8.1667	2.3333	5	1.8333	1
Total rank		9	10	8	6	7	6	4	11	3	5	2	1

**Table 4:** Evaluation results of fixed-dimensional multimodal functions.

		GA	PSO	GSA	TLBO	MVO	GWO	WOA	TSA	RSA	MPA	AVOA	DTBO
$F_{14}$	Mean	1.0487	3.5958	3.5613	0.9980	0.9980	3.6952	2.5698	8.6469	1.0477	4.1486	1.4863	0.9980
	Best	0.9980	0.9980	0.9980	0.9980	0.9980	0.9980	0.9980	1.9920	0.9980	1.0702	0.9980	0.9980
	Worst	1.9920	12.671	11.87	0.9980	0.9980	10.763	10.763	15.504	1.9920	11.735	10.763	0.9980
	std	0.2221	3.7879	2.7541	3.3E$-$06	5.7E$-$12	3.7310	2.9463	5.0513	0.2223	2.9540	2.1836	0
	Median	0.9980	1.9920	2.8917	0.9980	0.9980	2.9821	0.9980	11.717	0.9980	2.9821	0.9980	0.9980
	Rank	5	9	8	3	2	10	7	12	4	11	6	1
$F_{15}$	Mean	0.0154	0.0025	0.0024	0.0006	0.0026	0.0034	0.0008	0.0164	0.0003	0.0011	0.0004	0.0003
	Best	0.0008	0.0003	0.0009	0.0003	0.0003	0.0003	0.0003	0.0003	0.0003	0.0006	0.0003	0.0003
	Worst	0.0669	0.0204	0.0070	0.0012	0.0204	0.0204	0.0023	0.1103	0.0003	0.0019	0.0006	0.0003
	std	0.0162	0.0061	0.0014	0.0004	0.0061	0.0073	0.0005	0.0300	5.1E$-$11	0.0003	9.5E$-$05	2.5E$-$19
	Med	0.0143	0.0003	0.0022	0.0003	0.0007	0.0003	0.0007	0.0009	0.0003	0.001	0.0003	0.0003
	Rank	11	8	7	4	9	10	5	12	2	6	3	1
$F_{16}$	Mean	− 1.0316	− 1.0316	− 1.0316	− 1.0316	− 1.0316	− 1.0316	− 1.0316	− 1.0301	− 1.0316	− 1.0309	− 1.0316	− 1.0316
	Best	− 1.0316	− 1.0316	− 1.0316	− 1.0316	− 1.0316	− 1.0316	− 1.0316	− 1.0316	− 1.0316	− 1.0316	− 1.0316	− 1.0316
	Worst	− 1.0316	− 1.0316	− 1.0316	− 1.0316	− 1.0316	− 1.0316	− 1.0316	− 1	− 1.0316	− 1.0285	− 1.0316	− 1.0316
	std	4.8E$-$06	1.1E$-$16	1.0E$-$16	1.7E$-$06	5.5E$-$08	8.6E$-$09	4.0E$-$11	0.0071	2.4E$-$12	0.0009	8.8E$-$15	1.8E$-$16
	Median	− 1.0316	− 1.0316	− 1.0316	− 1.0316	− 1.0316	− 1.0316	− 1.0316	− 1.0316	− 1.0316	− 1.0313	− 1.0316	− 1.0316
	Rank	5	1	1	6	4	3	2	8	1	7	1	1
$F_{17}$	Mean	0.4660	0.7446	0.3979	0.3980	0.3979	0.3979	0.3979	0.3979	0.3979	0.4265	0.3979	0.3979
	Best	0.3979	0.3979	0.3979	0.3979	0.3979	0.3979	0.3979	0.3979	0.3979	0.3979	0.3979	0.3979
	Worst	1.7522	2.7912	0.3979	0.3982	0.3979	0.3979	0.3979	0.3982	0.3979	0.6306	0.3979	0.3979
	std	0.3027	0.7093	0	6.8E$-$05	6.6E$-$08	8.9E$-$07	7.3E$-$07	6.8E$-$05	0	0.0671	0	0
	Med	0.3979	0.3979	0.3979	0.3979	0.3979	0.3979	0.3979	0.3979	0.3979	0.4007	0.3979	0.3979
	Rank	8	9	1	6	2	4	3	5	1	7	1	1
$F_{18}$	Mean	7.3029	3	3	3	3	3	3	11.502	3	4.3828	3	3
	Best	3	3	3	3	3	3	3	3	3	3	3	3
	Worst	34.950	3	3	3	3	3	3	92.035	3	30.651	3	3
	std	10.544	3.0E$-$15	3.6E$-$15	1.7E$-$06	4.5E$-$07	1.5E$-$05	4.3E$-$05	26.200	5.5E$-$08	6.1828	1.8E$-$06	1.2E$-$15
	Median	3	3	3	3	3	3	3	3	3	3	3	3
	Rank	10	2	3	5	4	7	8	11	1	9	6	1
$F_{19}$	Mean	− 3.8626	− 3.8628	− 3.8628	− 3.8617	− 3.8628	− 3.8613	− 3.8604	− 3.8624	− 3.8628	− 3.8251	− 3.8628	− 3.8628
	Best	− 3.8628	− 3.8628	− 3.8628	− 3.8627	− 3.8628	− 3.8628	− 3.8628	− 3.8628	− 3.8628	− 3.8617	− 3.8628	− 3.8628
	Worst	− 3.8618	− 3.8628	− 3.8628	− 3.8549	− 3.8628	− 3.8550	− 3.8549	− 3.856	− 3.8628	− 3.6858	− 3.8628	− 3.8628
	std	0.0003	2.1E$-$15	2.00E$-$15	0.0023	2.1E$-$07	0.0026	0.0029	0.0015	2.2E$-$06	0.0416	2.5E$-$13	2.3E$-$15
	Med	− 3.8628	− 3.8628	− 3.8628	− 3.8624	− 3.8628	− 3.8628	− 3.8619	− 3.8627	− 3.8628	− 3.8406	− 3.8628	− 3.8628
	Rank	4	1	1	6	3	7	8	5	1	9	2	1
$F_{20}$	Mean	− 3.2283	− 3.2646	− 3.3220	− 3.2428	− 3.2743	− 3.2590	− 3.2499	− 3.2551	− 3.3220	− 2.7228	− 3.2863	− 3.3220
	Best	− 3.3216	− 3.3220	− 3.3220	− 3.3159	− 3.3220	− 3.3220	− 3.3220	− 3.3216	− 3.3220	− 3.0794	− 3.3220	− 3.3220
	Worst	− 2.9972	− 3.1376	− 3.322	− 3.0138	− 3.2023	− 3.084	− 3.0893	− 3.0895	− 3.3220	− 1.7526	− 3.2031	− 3.3220
	std	0.0782	0.0750	3.8E$-$16	0.0802	0.0599	0.0761	0.0839	0.0712	2.9E$-$08	0.3938	0.0559	4.4E$-$16
	Median	− 3.2366	− 3.322	− 3.322	− 3.2918	− 3.322	− 3.322	− 3.3181	− 3.2611	− 3.322	− 2.9059	− 3.322	− 3.322
	Rank	10	5	1	9	4	6	8	7	2	11	3	1
$F_{21}$	Mean	− 6.2602	− 5.6238	− 7.1941	− 6.8527	− 8.8855	− 9.3904	− 9.3854	− 5.9252	− 10.153	− 5.0552	− 10.153	− 10.153
	Best	− 9.7386	− 10.153	− 10.153	− 9.4150	− 10.153	− 10.153	− 10.153	− 10.13	− 10.153	− 5.0552	− 10.153	− 10.153
	Worst	− 2.3858	− 2.6305	− 2.6829	− 3.2427	− 5.0552	− 5.0552	− 5.0551	− 2.603	− 10.153	− 5.0552	− 10.153	− 10.153
	std	2.7111	2.8839	3.4577	2.0775	2.2527	1.862	1.8663	3.2356	7.3E$-$08	4.1E$-$07	1.0E$-$13	2.1E$-$15
	Med	− 7.0607	− 5.1008	− 10.153	− 7.314	− 10.153	− 10.153	− 10.151	− 4.9993	− 10.153	− 5.0552	− 10.153	− 10.153
	Rank	9	11	7	8	6	4	5	10	3	12	2	1
$F_{22}$	Mean	− 7.3719	− 6.3829	− 10.129	− 7.9498	− 8.4347	− 10.402	− 8.1085	− 6.8844	− 10.403	− 5.0877	− 10.403	− 10.403
	Best	− 9.9828	− 10.403	− 10.403	− 10.063	− 10.403	− 10.403	− 10.403	− 10.339	− 10.403	− 5.0877	− 10.403	− 10.403
	Worst	− 2.6768	− 2.7519	− 4.9295	− 4.0484	− 2.7659	− 10.402	− 1.8375	− 1.8328	− 10.403	− 5.0877	− 10.403	− 10.403
	std	1.9166	3.4696	1.2239	1.6734	2.7968	0.0004	3.0517	3.5094	1.0E$-$06	7.5E$-$07	1.0E$-$14	3.5E$-$15
	Median	− 7.8631	− 5.1083	− 10.403	− 8.3854	− 10.403	− 10.403	− 10.398	− 7.4911	− 10.403	− 5.0877	− 10.403	− 10.403
	Rank	9	11	5	8	6	4	7	10	3	12	2	1
$F_{23}$	Mean	− 6.3602	− 6.4208	− 10.287	− 8.0861	− 9.4619	− 10.536	− 8.5835	− 7.4150	− 10.536	− 5.1285	− 10.536	− 10.536
	Best	− 10.185	− 10.536	− 10.536	− 9.6908	− 10.536	− 10.536	− 10.536	− 10.481	− 10.536	− 5.1285	− 10.536	− 10.536
	Worst	− 2.3823	− 2.4217	− 5.5559	− 4.2682	− 5.1285	− 10.535	− 1.6765	− 2.4201	− 10.536	− 5.1285	− 10.536	− 10.536
	std	2.6086	3.8479	1.1137	1.6609	2.2049	0.0003	3.2621	3.4729	4.7E$-$07	2.1E$-$06	5.0E$-$15	2.8E$-$15
	Med	− 6.8883	− 3.8354	− 10.536	− 8.6793	− 10.536	− 10.536	− 10.534	− 10.290	− 10.536	− 5.1285	− 10.536	− 10.536
	Rank	11	10	5	8	6	4	7	9	3	12	2	1
Sum rank		82	67	39	63	46	59	60	89	21	96	28	10
Mean rank		8.2	6.7	3.9	6.3	4.6	5.9	6	8.9	2.1	9.6	2.8	1
Total rank		10	9	4	8	5	6	7	11	2	12	3	1

**Table 5:** Evaluation results of IEEE CEC 2017 objective functions $C_1$ to $C_{18}$.

		GA	PSO	GSA	TLBO	MVO	GWO	WOA	TSA	RSA	MPA	AVOA	DTBO
$C_1$	avg	9838.1	3966.4	297.27	2.0E+07	3.3E+05	8.5E+06	296.63	3408.0	156.74	2470.2	1286.7	100.00
	std	7142.0	5216.8	323.19	4.8E+06	1.2E+05	2.8E+07	319.69	4267.2	4.2E+04	313.50	421.05	578.82
	Rank	9	8	4	12	10	11	3	7	2	6	5	1
$C_2$	avg	5632.2	7083.8	7949.3	1.2E+04	314.27	461.55	216.36	220.06	201.05	201.77	201.25	200.00
	std	5026.0	2575.9	2480.6	7271.1	8461.6	8039.9	881.98	773.38	84.706	108.37	59.590	12.462
	Rank	9	10	11	12	7	8	5	6	2	4	3	1
$C_3$	avg	8726.3	301.28	1.1E+04	2.8E+04	1547.3	2.3E+04	1.1E+04	300.15	302.47	1512.9	909.45	300.00
	std	6770.9	2.3E$-$10	1826.6	1.0E+04	2212.3	4216.3	1865.2	0	56.73	29.63	14.99	1.2E$-$10
	Rank	8	3	10	12	7	11	9	2	4	6	5	1
$C_4$	avg	411.24	407.76	409.03	549.07	410.66	2400.3	408.11	406.27	403.46	405.62	402.33	400.00
	std	21.137	3.9187	3.3874	18.377	8.8624	495.94	3.3628	12.183	109.22	9.3258	4.9026	0.0687
	Rank	10	6	8	11	9	12	7	5	3	4	2	1
$C_5$	avg	518.51	515.04	557.94	742.32	516.28	902.00	558.92	523.44	532.17	514.44	513.35	510.00
	std	7.9381	7.5534	9.4864	41.477	7.1429	90.488	9.9242	12.120	67.685	27.985	16.614	4.4700
	Rank	6	4	9	11	5	12	10	7	8	3	2	1
$C_6$	avg	601.85	600.85	623.21	666.16	603.01	691.78	622.08	611.98	682.39	600.70	600.57	600.00
	std	0.0807	1.1129	10.276	49.802	1.0411	12.857	10.690	9.8197	41.598	1.6668	0.8165	7.4E$-$04
	Rank	5	4	9	10	6	12	8	7	11	3	2	1
$C_7$	avg	731.22	721.29	717.50	1280.6	733.15	1866.8	717.09	744.50	716.04	714.69	719.37	723.00
	std	8.3149	6.1026	1.7615	50.912	9.8460	109.27	1.8717	19.642	1.8781	5.0727	4.6767	4.6518
	Rank	8	6	4	11	9	12	3	10	2	1	5	7
$C_8$	avg	824.26	812.04	823.68	955.00	816.53	1070.5	823.59	824.90	829.74	812.60	809.23	809.00
	std	10.297	6.4292	5.3985	22.133	9.3912	50.750	5.5979	11.555	61.983	9.2155	6.4796	3.5578
	Rank	8	3	7	11	5	12	6	9	10	4	2	1
$C_9$	avg	913.14	902.37	900.41	6811.1	914.85	2.9E+04	902.21	946.36	4672.3	914.08	907.99	900.00
	std	17.270	7.0E−14	6.9E−15	1538.0	22.409	9978.6	0	126.19	2413.0	22.847	11.509	0.0193
	Rank	6	4	2	11	8	12	3	9	10	7	5	1
$C_{10}$	avg	1728.4	1472.2	2697.8	5291.0	1530.3	7484.5	2699.0	1867.3	2600.2	1411.2	1426.9	1440.0
	std	304.01	248.58	351.42	774.76	332.5	1542.4	344.85	348.83	489.35	40.891	100.08	161.60
	Rank	6	4	9	11	5	12	10	7	8	1	2	3
$C_{11}$	avg	1131.4	1111.2	1132.1	1276.0	1140.4	1923.3	1134.6	1183.7	1110.5	1112.1	1105.1	1100.0
	std	28.320	7.4178	12.650	47.856	61.623	2193.9	12.737	70.729	29.361	12.658	7.3046	1.4925
	Rank	6	4	7	11	9	12	8	10	3	5	2	1
$C_{12}$	avg	3.7E+04	1.5E+04	7.0E+05	2.2E+07	6.3E+05	1.8E+08	7.1E+05	2.0E+06	1637.2	1.5E+04	8226.7	1250.0
	std	4.1E+04	1.3E+04	4.9E+04	2.4E+07	1.3E+06	2.0E+09	4.8E+05	2.3E+06	233.16	3234.1	1550.0	64.192
	Rank	6	4	8	11	7	12	9	10	2	5	3	1
$C_{13}$	avg	1.1E+04	8623.9	1.1E+04	4.2E+05	9871.7	1.9E+08	1.1E+04	1.6E+04	1324.2	6853.0	4076.8	1310.0
	std	1.1E+04	6042.0	2392.3	1.5E+05	6566.9	1.6E+08	2444.4	1.3E+04	91.485	5075.5	2476.7	3.1148
	Rank	7	5	8	11	6	12	9	10	2	4	3	1
$C_{14}$	avg	7054.8	1486.6	7171.9	4.1E+05	3406.5	2.0E+06	7164.4	1514.5	1456.6	1454.9	1430.2	1400.0
	std	9713.5	49.535	1796.7	2.7E+05	2238.9	8.3E+06	1692.7	58.251	64.798	26.870	15.687	4.6010
	Rank	8	5	10	11	7	12	9	6	4	3	2	1
$C_{15}$	avg	9346.1	1716.2	1.8E+04	4.8E+04	3813.6	1.4E+07	1.8E+04	2248.3	1512.7	1581.1	1545.2	1500.0
	std	1.0E+04	342.51	6264.2	1.8E+04	4450.9	2.4E+07	6368.3	645.63	19.341	150.50	77.146	0.6144
	Rank	8	5	9	11	7	12	10	6	2	4	3	1
$C_{16}$	avg	1793.8	1860.6	2153.7	3513.3	1738.0	3004.2	2156.4	1732.1	1821.3	1734.5	1670.3	1600.0
	std	150.65	145.90	125.90	273.70	148.24	1426.7	122.66	151.72	276.80	137.49	72.936	1.1817
	Rank	6	8	9	12	5	11	10	3	7	4	2	1
$C_{17}$	avg	1750.3	1761.9	1865.1	2632.2	1764.1	4346.1	1861.7	1774.0	1832.2	1732.3	1725.0	1710.0
	std	46.452	56.813	124.00	226.70	37.236	380.86	124.57	41.396	204.39	41.375	26.497	11.404
	Rank	4	5	10	11	6	12	9	7	8	3	2	1
$C_{18}$	avg	1.6E+04	1.5E+04	8754.2	7.5E+05	2.6E+04	3.8E+07	8756.6	2.3E+04	1830.2	7464.9	4640.2	1800.0
	std	1.5E+04	1.4E+04	5915.6	4.3E+05	1.9E+04	5.6E+07	6084.8	1.7E+04	15.698	5099.5	2629.4	0.6111
	Rank	8	7	5	11	10	12	6	9	2	4	3	1

**Table 6:** Evaluation results of the IEEE CEC 2017 objective functions $C_{19}$ to $C_{30}$.

		GA	PSO	GSA	TLBO	MVO	GWO	WOA	TSA	RSA	MPA	AVOA	DTBO
$C_{19}$	avg	9731.0	2605.8	1.4E+04	6.1E+05	9892.8	2.3E+06	4.5E+04	2926.0	1925.8	1952.9	1930.9	1900.0
	std	7858.3	2581.0	2.2E+04	6.6E+05	7399.9	1.8E+07	2.2E+04	2196.8	33.850	62.668	31.866	0.5177
	Rank	7	5	9	11	8	12	10	6	2	4	3	1
$C_{20}$	avg	2060.5	2098.1	2280.2	2880.4	2084.0	3805.4	2277.5	2090.0	2493.9	2025.1	2026.6	2020.0
	std	68.762	75.043	92.511	245.57	59.512	532.00	97.367	57.113	286.71	28.694	19.776	11.056
	Rank	4	7	9	11	5	12	8	6	10	2	3	1
$C_{21}$	avg	2301.9	2281.0	2364.7	2580.4	2320.2	2580.6	2371.7	2255.0	2328.3	2233.5	2225.6	2200.0
	std	50.749	65.303	32.539	71.887	8.0010	217.34	33.053	72.501	78.370	50.003	37.064	23.769
	Rank	6	5	9	11	7	12	10	4	8	3	2	1
$C_{22}$	avg	2307.9	2312.5	2308.9	7208.1	2316.1	1.4E+04	2301.3	2308.6	3534.3	2287.8	2290.5	2280.0
	std	2.8287	76.143	0.0826	1545.5	19.061	1188.7	0.0846	13.699	972.08	15.320	30.114	44.375
	Rank	5	8	7	11	9	12	4	6	10	2	3	1
$C_{23}$	avg	2634.3	2632.1	2751.7	3124.3	2631.3	3826.7	2751.3	2630.7	2730.2	2612.6	2622.4	2610.0
	std	15.619	10.570	45.031	96.724	9.5706	250.98	46.255	10.124	284.18	4.9156	4.5501	4.6847
	Rank	7	6	10	11	5	12	9	4	8	2	3	1
$C_{24}$	avg	2764.1	2696.7	2748.1	3342.0	2742.6	3480.6	2753.4	2740.3	2701.6	2626.3	2574.3	2520.0
	std	17.772	124.54	6.5110	189.89	9.8817	250.01	6.4110	76.566	86.826	95.951	70.877	43.496
	Rank	10	4	8	11	7	12	9	6	5	3	2	1
$C_{25}$	avg	2955.5	2929.2	2943.2	2920.6	2940.6	3920.2	2950.1	2932.1	2936.3	2923.4	2917.2	2900.0
	std	23.363	30.274	17.686	21.231	27.954	288.36	18.086	28.773	23.595	14.650	7.8934	0.5732
	Rank	11	5	9	3	8	12	10	6	7	4	2	1
$C_{26}$	avg	3110.6	2952.4	3.4E+04	7886.1	3222.1	7105.4	3454.4	2904.0	3462.6	3125.2	2991.1	2850.0
	std	396.58	300.50	752.69	1099.0	492.04	3364.5	723.62	43.795	699.89	337.31	222.65	111.56
	Rank	5	3	12	11	7	10	8	2	9	6	4	1
$C_{27}$	avg	3126.2	3121.7	3273.6	3419.8	3114.9	4827.4	3271.4	3098.9	3149.0	3116.0	3100.4	3090.0
	std	21.882	29.347	48.343	98.368	24.965	736.161	48.582	3.303	25.373	23.812	12.238	0.5212
	Rank	7	6	10	11	4	12	9	2	8	5	3	1
$C_{28}$	avg	3325.4	3330.3	3472.5	3413.4	3392.5	5107.4	3465.8	3217.9	3413.1	2303.3	2709.2	3100.0
	std	150.94	141.55	39.174	140.03	117.32	374.55	40.862	131.96	153.87	140.48	71.968	7.7E$-$05
	Rank	5	6	11	9	7	12	10	4	8	1	2	3
$C_{29}$	avg	3260.6	3205.6	3452.2	4562.6	3196.3	8920.8	3463.9	3216.3	3218.5	3216.4	3191.4	3150.0
	std	97.691	60.193	197.37	583.47	51.265	1691.3	206.57	61.883	128.70	67.701	40.254	15.064
	Rank	8	4	9	11	3	12	10	5	7	6	2	1
$C_{30}$	avg	5.4E+05	3.5E+05	1.3E+06	4.0E+06	3.0E+05	1.9E+07	9.4E+05	4.2E+05	3.1E+05	3.0E+05	1.5E+05	3410.0
	std	7.2E+05	6.1E+05	4.1E+05	1.9E+06	6.3E+05	1.59E+08	4.1E+05	6.4E+05	5.3E+05	2.6E+04	1.3E+04	31.986
	Rank	8	6	10	11	4	12	9	7	5	3	2	1
Sum rank		211	160	252	323	202	351	240	188	177	112	84	40
Mean rank		7.0333	5.3333	8.4	10.767	6.7333	11.7	8	6.2667	5.9	3.7333	2.8	1.3333
Total rank		8	4	10	11	7	12	9	6	5	3	2	1

**Table 7:** *p* values from Wilcoxon sum rank test.

Compared algorithms	Test function type			
	Unimodal	High-multimodal	Fixed-multimodal	IEEE CEC2017
DTBO vs. GA	1.01E$-$24	1.97E$-$21	0.005203	2.06E$-$16
DTBO vs. PSO	1.01E$-$24	1.97E$-$21	1.23E$-$13	5.68E$-$18
DTBO vs. GSA	6.24E$-$18	2.70E$-$18	4.05E$-$05	1.21E$-$12
DTBO vs. TLBO	1.01E$-$24	6.98E$-$15	9.67E$-$18	4.68E$-$16
DTBO vs. MVO	1.01E$-$24	1.97E$-$21	3.88E$-$12	1.61E$-$19
DTBO vs. GWO	5.71E$-$24	5.34E$-$16	3.88E$-$07	1.37E$-$10
DTBO vs. WOA	6.91E$-$24	0.003366	0.010621	3.82E$-$07
DTBO vs. TSA	1.01E$-$24	1.31E$-$20	1.44E$-$34	6.32E$-$25
DTBO vs. MPA	1.23E$-$09	0.550347	1.16E$-$10	5.34E$-$08
DTBO vs. RSA	0.004063	4.33E$-$08	1.37E$-$30	6.33E$-$28
DTBO vs. AVOA	7.03E$-$05	6.42E$-$04	0.005203	3.13E$-$02

**Table 8:** Performance of optimization algorithms in pressure vessel design.

Algorithms	Optimum variables				Optimum cost
	*h*	*l*	*t*	*b*	
DTBO	0.778635	0.385303	40.34282	199.5782	5885.355
AVOA	0.778949	0.385038	40.35999	199.1993	5891.422
RSA	0.840909	0.419378	43.42455	161.7172	6040.794
MPA	0.815064	0.445655	42.24451	176.7981	6119.433
TSA	0.788364	0.389911	40.84104	200.2000	5922.697
WOA	0.789199	0.389678	40.85395	200.2000	5926.513
GWO	0.819006	0.441004	42.43535	178.0534	5928.544
MVO	0.856754	0.424026	44.38794	158.4219	6049.427
TLBO	0.828244	0.423385	42.29410	185.9678	6176.079
GSA	1.099967	0.962004	49.98904	171.6986	11623.14
PSO	0.762178	0.404753	40.98030	199.5860	5927.478
GA	1.113869	0.918407	45.03642	182.0029	6591.333

**Table 9:** Statistical results of optimization algorithms in the design of pressure vessels.

Algorithms	Best	Mean	Worst	Std. Dev.	Median
DTBO	5885.3548	5887.8210	5897.107	21.02136	5889.619
AVOA	5891.4220	5891.4240	5891.738	31.16894	5894.294
RSA	6040.7940	6048.0930	6051.960	31.23574	6046.182
MPA	6119.4330	6127.3280	6138.652	38.30140	6125.140
TSA	5922.6970	5898.0470	5902.933	28.98210	5896.829
WOA	5926.5130	5902.1340	5905.239	13.93506	5901.258
GWO	5928.5440	6075.9400	7407.905	66.73857	6427.669
MVO	6049.4270	6488.9700	7263.975	327.5960	6409.002
TLBO	6176.0790	6338.1550	6524.083	126.8370	6329.696
GSA	11623.140	6852.8620	7172.184	5801.053	6849.947
PSO	5927.4780	6275.2860	7018.367	497.0215	6123.699
GA	6591.3330	6655.9520	8019.857	658.7072	7599.671

**Table 10:** Performance of optimization algorithms in the design of welded beams.

Algorithms	Optimum variables				Optimum cost
	*h*	*l*	*t*	*b*	
DTBO	0.205730	3.470500	9.036600	0.205730	1.724900
AVOA	0.205936	3.473962	9.045661	0.205936	1.726578
RSA	0.144825	3.517514	8.934025	0.211832	1.674273
MPA	0.218678	3.513750	8.881413	0.225135	1.867986
TSA	0.205769	3.478321	9.044835	0.206017	1.729384
WOA	0.205884	3.478878	9.046000	0.206435	1.730721
GWO	0.197608	3.318376	10.00800	0.201596	1.824323
MVO	0.205817	3.475574	9.049972	0.205915	1.729194
TLBO	0.204900	3.539827	9.013294	0.210235	1.762968
GSA	0.147245	5.496235	10.01000	0.217943	2.177546
PSO	0.164335	4.036574	10.01000	0.223871	1.878014
GA	0.206693	3.639508	10.01000	0.203452	1.840211

**Table 11:** Statistical results of optimization algorithms in the design of welded beams.

Algorithms	Best	Mean	Worst	Std. Dev.	Median
DTBO	1.724910	1.728057	1.730148	0.004332	1.727332
AVOA	1.726578	1.728851	1.729280	0.005128	1.727550
RSA	1.674273	1.705118	1.763902	0.017442	1.728144
MPA	1.867986	1.893952	2.018394	0.007968	1.885424
TSA	1.729384	1.730591	1.730826	0.000287	1.730549
WOA	1.730721	1.731893	1.732330	0.001161	1.731852
GWO	1.824323	2.236462	3.056641	0.325421	2.250856
MVO	1.729194	1.734452	1.746456	0.004881	1.732185
TLBO	1.762968	1.822671	1.878577	0.027619	1.825149
GSA	2.177546	2.551258	3.011943	0.256565	2.501997
PSO	1.878014	2.125086	2.326525	0.034916	2.102834
GA	1.840211	1.367289	2.040862	0.139871	1.941088

## Discussion

The optimization mechanism in metaheuristic algorithms is based on a random search in the problem solving space. An algorithm will be able to search accurately and effectively in the search space when it scans the various search spaces and around promising areas. This fact means that the power of exploration in the global search and the power of exploitation in the local search have a significant impact on the performance of optimization algorithms. The DTBO update process has three different phases with the aim of providing a global and a local search. The first phase of the update based on “training by the driving instructor” scans different parts of the search space according to the ability to explore. The second phase of the implementation of DTBO also increases the DTBO exploration power by making sudden changes in the population position. The third phase of DTBO, called the “practice”, leads to local search and increases the exploitation ability of DTBO. The important thing about exploration and exploitation is that, in the initial iterations, priority is given to global search, so that the algorithm can scan different parts of the search space. The update equations in the second and third phases are designed to make larger changes to the population in the initial iterations. As a result, in initial iterations, the DTBO population displacement range is larger, leading to its effective exploration. As the replication of the algorithm increases, it is important that the algorithm moves to better areas in the search space and scans the search space around promising solutions in smaller steps. The update equations in the second and third phases are adjusted to provide smaller changes to the population by increasing the iterations of the algorithm and to converge to the optimal solution with smaller and more precise steps. These strategies in the process of updating the members of the population in DTBO have led to the proposed approach, which in addition to the high capability in exploration and exploitation, also has a good balance between these two capabilities. Because they have only one optimal solution, unimodal objective functions are suitable options for measuring the exploitation power of optimization algorithms in convergence towards global optimal. The results of optimization of the unimodal functions show that DTBO has a high exploitation capability in local search. Therefore, this algorithm has converged precisely to the global optimum to solve functions $F_1$ to $F_6$. High-dimensional multimodal objective functions are suitable options for evaluating the exploration power of optimization algorithms in identifying the main optimal area because they have many local optimal areas in the search space. The results obtained from the optimization of the functions $F_8$ to $F_{13}$ indicate the high exploration ability of DTBO. In the case of functions $F_9$ and $F_{11}$, after identifying the optimal area, it also converges to the global optimal. Fixed-dimensional multimodal objective functions, because they have fewer local optimal solutions (compared to functions $F_8$ to $F_{13}$), are good options for analyzing the ability of optimization algorithms to maintain the balance between exploration and exploitation. The optimization results of functions $F_{14}$ to $F_{23}$ show that DTBO can provide optimal solutions for these optimization problems by creating a proper balance between exploration and exploitation.

The IEEE CEC2017 benchmark functions are also suitable to further challenge DTBO in solving more complex optimization problems. The results obtained from the optimization of the functions $C_1$ to $C_{30}$ indicate the high capability of the proposed DTBO to solve complex optimization problems.

## DTBO for real-world applications

In this section, the ability of DTBO to provide the optimal solution for real-world optimization applications is challenged. For this purpose, DTBO and competing algorithms have been implemented in two optimization challenges, pressure vessel design and welded beam design.

### Pressure vessel design

**Figure 4:**
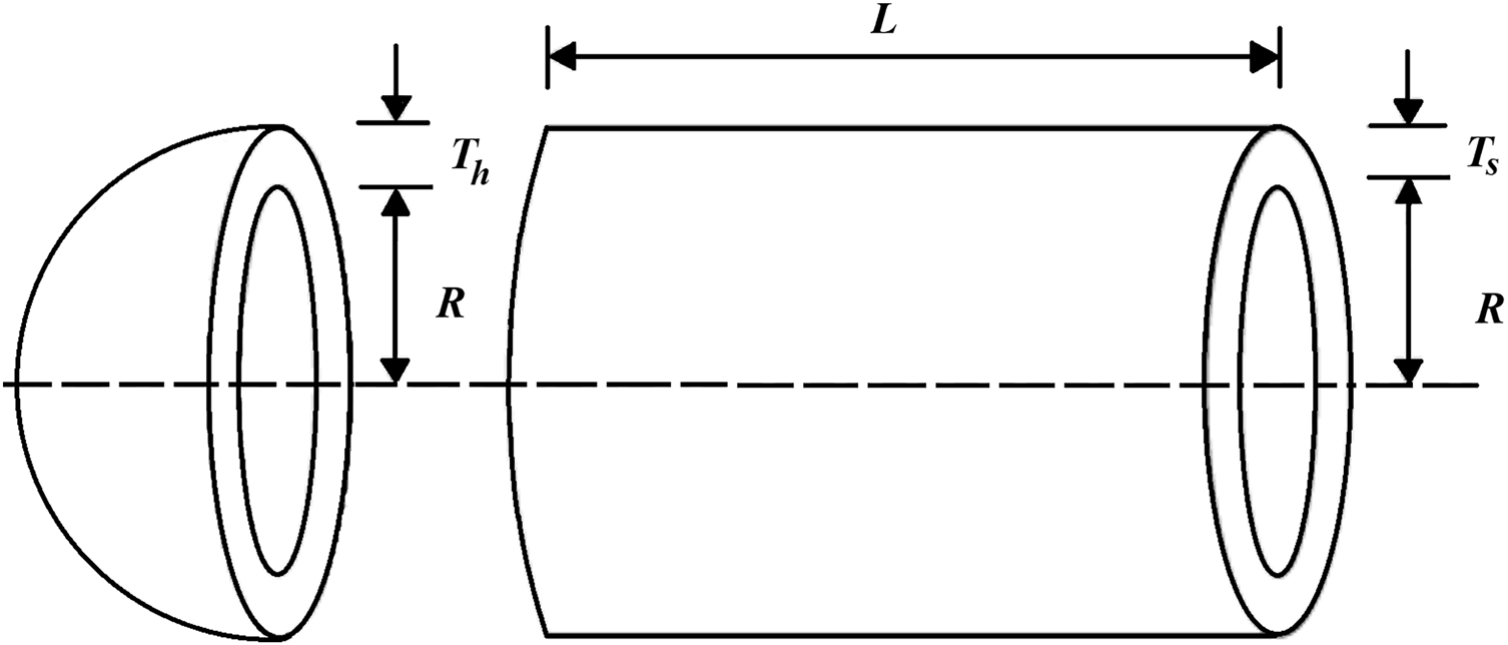
Schematic of pressure vessel design.

**Figure 5:**
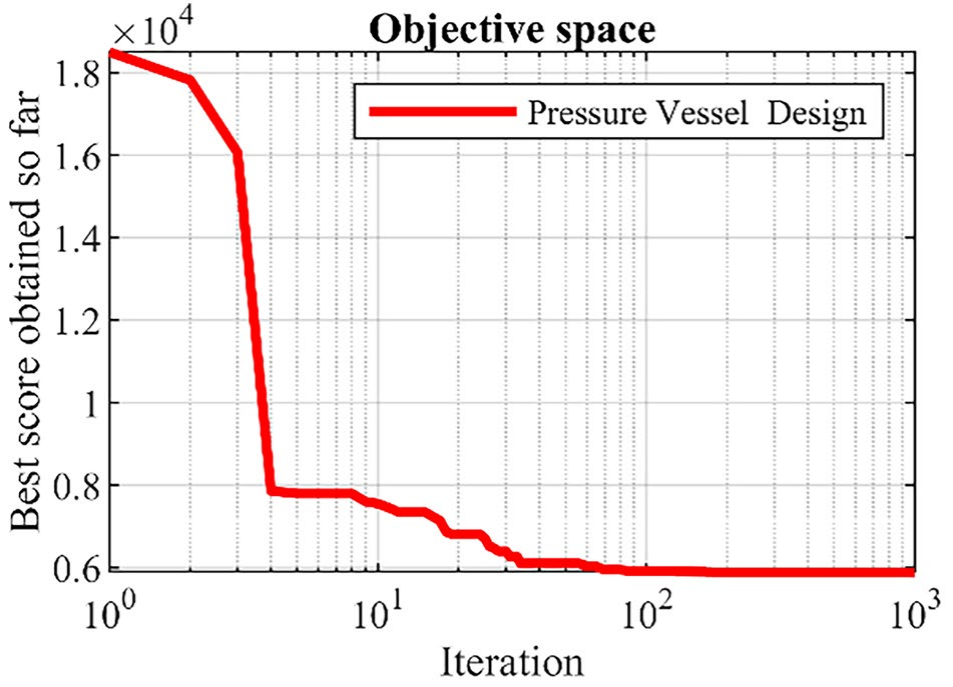
DTBO’s performance convergence curve in the design of a pressure vessel.

Pressure vessel design is a real-world optimization theme aimed at minimizing design costs, a schematic of which is shown in Fig. 4^56^. The results of the implementation of the proposed DTBO and competitor algorithms in this challenge are reported in Tables 8 and  9. Based on the optimization results, DTBO has provided the solution to this problem with the values of the design variables equal to (0.7786347, 0.3853025, 40.34282, 199.5782) and the value of the objective function equal to 5885.3548. Analysis of the simulation results shows that DTBO has performed better than competitor algorithms in providing solutions and statistical indicators. The DTBO convergence curve while finding the solution to the pressure vessel design problem is shown in Fig. 5.

### Welded beam design

**Figure 6:**
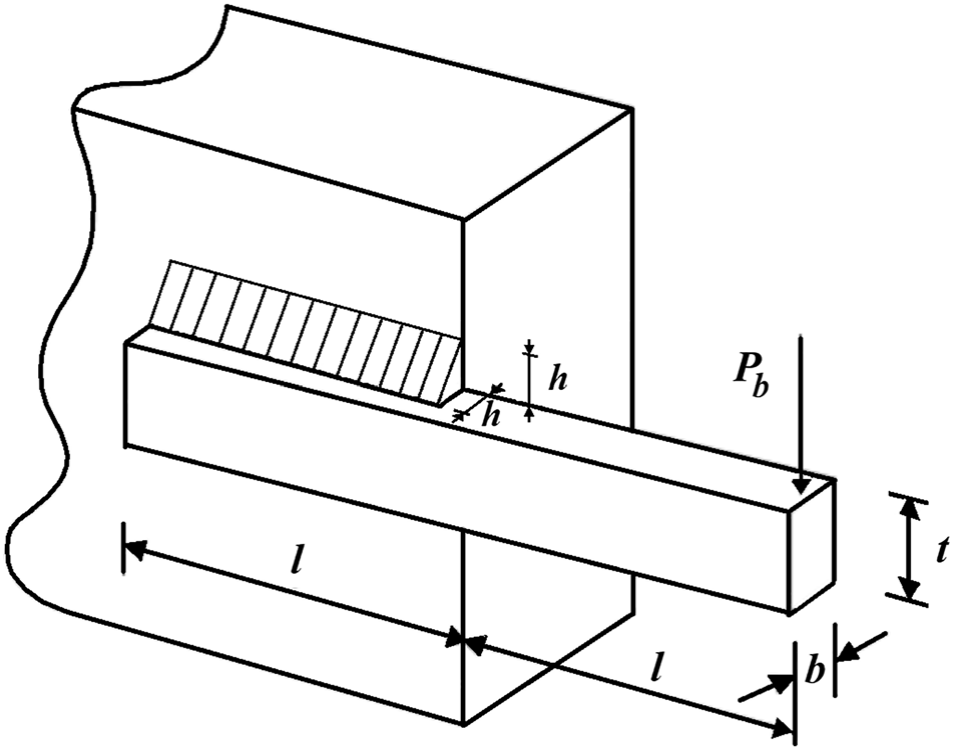
Schematic of welded beam design.

**Figure 7:**
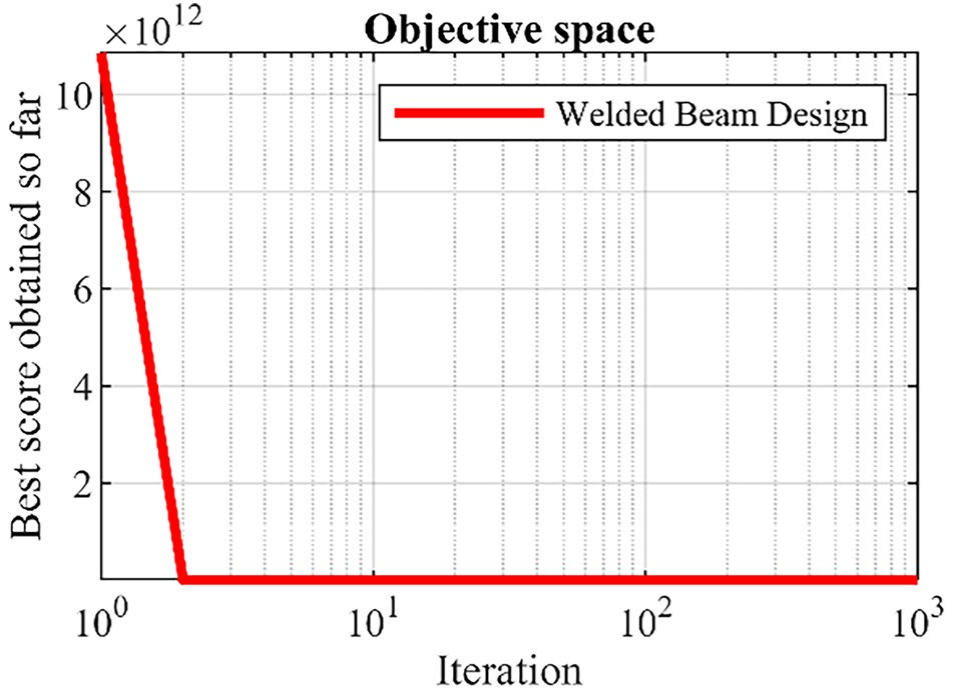
DTBO performance convergence curve for the welded beam design.

Welded beam design is an engineering optimization problem aimed at reducing the fabrication cost, the schematic is shown in Fig. 6^13^. The optimization results of this design using DTBO and competitor algorithms are presented in Table 10 and Table 11. The results show that DTBO has provided the solution to this problem with the values of the design variables equal to (0.20573, 3.4705, 9.0366, 0.20573) and the value of the objective function equal to 1.7249. What can be deduced from the simulation results is that DTBO has provided a more efficient solution to this problem compared to competitor algorithms by providing a better solution and better statistical indicators. The DTBO convergence curve while finding the solution to the design problem of welded beams is shown in Fig. 7.

## Conclusion and future works

This paper introduced a new stochastic human-based algorithm called Driving Training-Based Optimization (DTBO). The process of learning to drive in a driving school is the fundamental inspiration of the DTBO design. DTBO was mathematically modeled in three phases: (i) training by the driving instructor, (ii) patterning of students from instructor skills, and (iii) practice. Furthermore, we have shown the performance of DTBO in optimizing fifty-three objective functions of a group of unimodal, high-dimensional, fixed-dimensional multimodal, and IEE CEC2017. The results obtained from the implementation of DTBO in the objective functions $F_1$ to $F_{23}$ showed that DTBO has a high ability to exploit, explore, and balance them to perform powerfully in the optimization process.

The optimization results of the functions $C_1$ to $C_{30}$ showed the acceptable ability of DTBO to solve complex optimization problems.

To analyze the performance of DTBO, we compared its results with the performance of 11 well-known algorithms. A comparison of DTBO performance against competitor algorithms showed that the proposed DTBO, with better results, is more effective in optimizing and achieving optimal solutions and is much more competitive than the algorithms compared.

The use of DTBO in addressing two engineering design issues demonstrated the effective ability of the proposed approach in solving real-world applications. The authors offer several research pathways for future studies, including the development of binary and multi-objective versions of DTBO, which are among the particular study potentials of this paper. The application of DTBO in optimization problems in various sciences and real-world optimization challenges are other perspectives on the study of the proposed approach.

Although DTBO has provided acceptable results in solving the problems studied in this paper, there are some limitations to this method in other applications. The authors do not in any way claim that DTBO is the best optimizer in solving optimization problems because according to the concept of the NFL theorem, such a hypothesis is completely and definitively rejected. Therefore, DTBO may not be effective in solving some optimization applications. Furthermore, the main limitation of any metaheuristic algorithm, including DTBO, is that there is always the possibility that new optimization approaches may be developed in the future that perform better in the handling of optimization applications.

## Data Availability

All data generated or analyzed during this study are included directly in the text of this submitted manuscript. There are no additional external files with datasets.

## Code availability

The source code of the DTBO algorithm is available at: https://uk.mathworks.com/matlabcentral/fileexchange/110755-driving-training-based-optimization.
